# Uptake of l-Alanine and Its Distinct Roles in the Bioenergetics of Trypanosoma cruzi

**DOI:** 10.1128/mSphereDirect.00338-18

**Published:** 2018-07-18

**Authors:** Richard M. B. M. Girard, Marcell Crispim, Mayke Bezerra Alencar, Ariel Mariano Silber

**Affiliations:** a Department of Parasitology, Laboratory of Biochemistry of Tryps - LaBTryps, Institute of Biomedical Sciences, University of São Paulo, Cidade Universitária, Butantã, São Paulo, São Paulo, Brazil; University at Buffalo; University of Glasgow; University of Edinburgh; University of Georgia

**Keywords:** Chagas disease, l-alanine metabolism, l-alanine uptake, Trypanosoma cruzi, bioenergetics

## Abstract

Amino acids participate in several critical processes in the biology of trypanosomatids, such as osmoregulation, cell differentiation, and host cell invasion. Some of them provide reducing power for mitochondrial ATP synthesis. It was previously shown that alanine, which is formed mainly by the amination of pyruvate, is a metabolic end product formed when parasites are replicating in a medium rich in glucose and amino acids. It was shown as well that this amino acid can also be used for the regulation of cell volume and resistance to osmotic stress. In this work, we demonstrate that, despite it being an end product of its metabolism, Trypanosoma cruzi can take up and metabolize l-Ala through a low-specificity nonstereoselective active transport system. The uptake was dependent on the temperature in the range between 10 and 40°C, which allowed us to calculate an activation energy of 66.4 kJ/mol and estimate the number of transporters per cell at ~436,000. We show as well that, once taken up by the cells, l-Ala can be completely oxidized to CO_2_, supplying electrons to the electron transport chain, maintaining the electrochemical proton gradient across the mitochondrial inner membrane, and supporting ATP synthesis in T. cruzi epimastigotes. Our data demonstrate a dual role for Ala in the parasite’s bioenergetics, by being a secreted end product of glucose catabolism and taken up as nutrient for oxidative mitochondrial metabolism.

**IMPORTANCE** It is well known that trypanosomatids such as the etiological agent of Chagas’ disease, Trypanosoma cruzi, produce alanine as a main end product of their energy metabolism when they grow in a medium containing glucose and amino acids. In this work, we investigated if under starvation conditions (which happen during the parasite life cycle) the secreted alanine could be recovered from the extracellular medium and used as an energy source. Herein we show that indeed, in parasites submitted to metabolic stress, this metabolite can be taken up and used as an energy source for ATP synthesis, allowing the parasite to extend its survival under starvation conditions. The obtained results point to a dual role for Ala in the parasite’s bioenergetics, by being a secreted end product of glucose catabolism and taken up as nutrient for oxidative mitochondrial metabolism.

## Abstract (precis)

It is well known that trypanosomatids such as the etiological agent of Chagas’ disease, Trypanosoma cruzi, produce alanine as a main end product of their energy metabolism when they grow in a medium containing glucose and amino acids. In this work, we investigated if under starvation conditions (which happen during the parasite life cycle) the secreted alanine could be recovered from the extracellular medium and used as an energy source. Herein we show that indeed, in parasites submitted to metabolic stress, this metabolite can be taken up and used as an energy source for ATP synthesis, allowing the parasite to extend its survival under starvation conditions. The obtained results point to a dual role for Ala in the parasite’s bioenergetics, by being a secreted end product of glucose catabolism and taken up as nutrient for oxidative mitochondrial metabolism.

## INTRODUCTION

Trypanosoma cruzi, the etiological agent of Chagas’ disease or American trypanosomiasis, is a quite unique organism in terms of its metabolism and bioenergetics (1, 2). This protist experiences a myriad of environmental conditions during its complex life cycle, which occur inside the entire digestive tube of triatomine insect vectors, the blood of more than 100 species of mammals, and the cytosol of (potentially) every mammalian nucleated cell in every tissue and organ (3). As a consequence of its transit through all these different environments, T. cruzi faces different conditions, varying in terms of the availability of nutrients, especially inside the insect vector, where T. cruzi could be confronted by severe nutritional stress (4, 5). Therefore, T. cruzi has to be equipped with a set of transporters and enzymes able to take up and metabolize the metabolites available in each one of these environments (2, 6). Among such different metabolites, it was consistently shown that several amino acids can be used as an energy source: Pro, Asp, His, Glu, Asn, Gln, Leu, and Ile (1, 6–14). Beyond their role in the parasite bioenergetics and protein synthesis, amino acids are involved in various critical biological functions in T. cruzi, such as cell differentiation, resistance to different forms of oxidative stress and starvation, infection of the mammalian host cells, and proliferation in the intracellular environment (2, 11, 12, 15, 16).

Ala, together with succinate, is one of the end products of the metabolism of glucose by epimastigotes, the parasite’s form living in the digestive tube of the insect vector, and as such, the main intracellular and secreted amino acid (17–19). Ala is the product of the reversible amination of pyruvate. Under conditions of excess of NH_4_^+^, Ala can be produced through an Ala dehydrogenase or the concerted action of an NAD-linked Glu dehydrogenase and aminotransferases that accept pyruvate as a substrate (20–23). Thus, Ala production might be also linked to reoxidation of glycolytically produced NADH, even under aerobic conditions (17, 24, 25). Interestingly, both intracellular and secreted pools of Ala are produced separately and were shown to be compartmentalized (18). Notably, early studies suggested that, despite being an end product of the metabolism, Ala can be metabolized by T. cruzi since it was able to trigger O_2_ consumption (10). Indeed, depending on the relative quantity of substrates and products, Ala could be reconverted into pyruvate by the same aminotransferases or Ala dehydrogenases that produce it (20, 25).

Another relevant role involving formation, influx, and efflux of Ala is its participation as part of the response to osmotic stress in T. cruzi (26–28). Notwithstanding its biological significance, Ala uptake and oxidation have not yet been characterized in T. cruzi. In this work, we biochemically describe in this organism a single Ala transport system as well as the mitochondrial oxidation of the amino acid through the evaluation of bioenergetics parameters. Ala, a multifunctional metabolite, depending on the metabolic conditions, can be a metabolic end product or can be a substrate to feed electrons into the respiratory chain for ATP production.

## RESULTS

### l-Ala uptake in T. cruzi epimastigotes.

**FIG 1:**
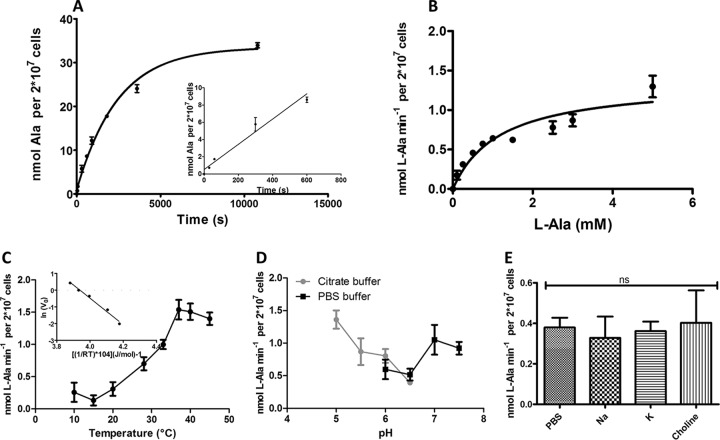
l-Ala uptake characterization. (A) Time course of l-Ala incorporation by the epimastigotes of Trypanosoma cruzi. The incorporation of 5 mM l-Ala traced with a radiolabeled amino acid was followed up as described in Materials and Methods. (Inset) l-Alanine was incorporated into the cells in a nearly linear manner for 3 min. All of the assays were performed in technical triplicate, and the data correspond to a representative of four independent biological experiments. (B) Effect of l-alanine concentration on l-Ala uptake as described in Materials and Methods. All of the assays were performed in technical triplicate, and the data correspond to a representative of four independent biological experiments. (C) Effect of temperature on l-Ala uptake. Epimastigotes of T. cruzi were incubated in the presence of l-Ala at different temperatures ranging between 10 and 45°C, and the *V*_0_ was measured. (Inset) Arrhenius plot from which the apparent energy of activation (*E*_*a*_) and Arrhenius constant (*A*) were calculated. All of the assays were performed in technical triplicate, and the data correspond to mean values from four independent biological experiments. (D) pH dependence of l-Ala transport in epimastigotes of T. cruzi. *V*_0_ was measured at pH values ranging between 5.0 and 7.5. For the range of pH values between 5.0 and 6.5, citrate buffer was used, whereas for the range of pH values between 6 and 7.5, PBS buffer was used. All of the assays were performed in technical triplicate, and the data correspond to a representative of three independent biological experiments. (E) The effect of cations Na^+^ and K^+^ on l-Ala transport. *V*_0_ was measured in cells resuspended in PBS (145 mM Na^+^ and 4.5 mM K^+^) or in phosphate buffers containing only Na^+^ (149.5 mM) or K^+^ (149.5 mM) as cations. Choline (139.7 mM) was used as the control. These data were analyzed using one-way analysis of variance (ANOVA) and Tukey’s posttest. No statistical differences (ns) were observed at *P* < 0.05.

To characterize the l-Ala transport system, we initially performed a time course assay for the uptake of l-Ala at a presumably saturating substrate concentration. For this, we incubated the parasites in the presence of 5 mM l-Ala and monitored the internalization of the amino acid over time. The obtained data could be fitted by an exponential decay function (*r*^2^ = 0.97), as expected for the uptake of metabolites mediated by a transport system (Fig. 1A). Given that the transported l-Ala increased in an approximately linear way for up to 3 min (*r*^2^ = 0.96 [Fig. 1A, inset]) the incubation time to measure the initial velocity (*V*_0_) of l-Ala transport was set to 1 min. In order to calculate the kinetic parameters of the l-Ala uptake process, *V*_0_ was measured as a function of the l-Ala extracellular concentration. A classical Michaelis-Menten hyperbolic function approached the data (*r*^2^ = 0.89), allowing the calculation of both kinetic parameters *V*_max_ and *K*_m_, which were 1.86 ± 0.3 nmol·min^−1^ per 20 × 10^6^ cells and 1.81 0.6 ± mM, respectively (Fig. 1B; see Fig. S1 and Table S1 in the supplemental material).

10.1128/mSphereDirect.00338-18.2 FIG S1  Effect of l-alanine concentration on l-Ala uptake as described in Materials and Methods. The data represent the four biological assays (A B, C, and D). Download FIG S1, TIF file, 0.1 MB. Copyright © 2018 Girard et al. 2018 Girard et al. This content is distributed under the terms of the Creative Commons Attribution 4.0 International license.

10.1128/mSphereDirect.00338-18.5 TABLE S1  Kinetic values corresponding to each experiment as described in Materials and Methods. Four measurements of *V*_0_ were made in triplicate as a function of Ala concentration. Each measurement was fitted to a hyperbolic function according to the Michaelis-Menten kinetic model (Fig. S1). From these nonlinear regression data, the parameters *K*_*m*_ and *V*_max_ were obtained for each experiment (named A, B, C, and D). The parameters reported correspond to the mean values and standard errors from these four biological replicates. Download TABLE S1, DOCX file, 0.01 MB. Copyright © 2018 Girard et al. 2018 Girard et al. This content is distributed under the terms of the Creative Commons Attribution 4.0 International license.

To determine the transporter specificity, we evaluated the ability of other amino acids as competitors. Short-chain amino acids, such as l-Gly and l-Ser, strongly inhibited l-Ala transport (inhibition of 76% and 84%, respectively). l-Cys and l-Pro also inhibited the l-Ala uptake by the cells, but to a lesser extent (inhibition of 53% and 33%, respectively). Remarkably, a 10-fold excess of d-Ala inhibited l-Ala uptake by 43.7%. All other amino acids tested as possible competitors of l-Ala uptake showed only a weak inhibition pattern if any (Table 1).

**TABLE 1:** Percentage of inhibition of l-Ala uptake when the transport assay was performed with parasites in the presence of 10-fold excess of competitor amino acid

Competitor	% of l-Ala uptake inhibition
d-Alanine	44.9 ± 3.5
Glycine	76 ± 10
Serine	84 ± 7.5
Cysteine	53 ± 9
Proline	33 ± 8.2
Aspartate	10 ± 7.4
Glutamate	10 ± 1.7
Glutamine	9.5 ± 7.4

### Thermodynamic analysis of l-Ala transport.

The effect of temperature on l-Ala uptake was evaluated by measuring *V*_max_ (assuming that *V*_max_ is equivalent to *V*_0_ at a saturating l-Ala concentration [5 mM]) at temperatures ranging from 10 to 45°C. As expected, an exponential increase of *V*_0_ was observed as a function of the temperature between 15 and 37°C, while in the range of 40 to 45°C, no velocity increases were observed (Fig. 1C). The changes in *V*_0_ in the exponential region of the curve were used to compute *Q*_10_, which was 2.47. *Q*_10_ is the ratio of the velocity of a reaction at a given temperature to that of the same reaction at a temperature 10°C lower. The invariant *V*_0_ value obtained for temperatures above 40°C can be attributed to the fact that temperature-dependent protein denaturation would be compensating any increase in activity. The *V*_0_ measurements made between 15 and 37°C were also used to calculate the energy of activation (*E*_*a*_) from an Arrhenius plot, which resulted to be 66.4 ± 9 kJ/mol (Fig. 1C, inset). Additionally, from the Arrhenius equation, it was possible to calculate the approximate number of transporters being used for the substrate uptake. The value 1.38 attomol per cell (see Text S1 in the supplemental material) could be estimated, which would be equivalent to approximately 4.36 × 10^5^ transporters per cell.

10.1128/mSphereDirect.00338-18.1 TEXT S1  Details of thermodynamic calculations. Download TEXT S1, DOCX file, 0.02 MB. Copyright © 2018 Girard et al. 2018 Girard et al. This content is distributed under the terms of the Creative Commons Attribution 4.0 International license.

### External ion sensitivity and driving force of l-Ala uptake.

To advance the biochemical characterization of the l-Ala transporter, we were interested in measuring the influence of H^+^, Na^+^, and K^+^ on its uptake. The H^+^ dependence of l-Ala uptake was analyzed by measuring *V*_0_ at different pH values in the range from 5 to 7.5. Our data exhibited a maximum value for *V*_0_ at pH 5.0, with a sharp decrease in the range between 6.0 and 6.5. Interestingly, at pH values between 7.0 and 7.5, we observed a significant increase, reaching levels close to those observed at an acidic pH (Fig. 1D). Next, we evaluated the effect of Na^+^ and K^+^ on l-Ala uptake. The transport was measured in modified phosphate-buffered saline (PBS) buffers, enriched in Na^+^ (K^+^ excluded), in K^+^ (Na^+^ excluded), regular PBS, or PBS in which the ionic strength was adjusted with choline (avoiding supplementation with Na^+^ or K^+^). None of the conditions affected l-Ala uptake (Fig. 1E).

To determine if l-Ala uptake involves active transport, the influence of the intracellular ATP levels on *V*_0_ was initially evaluated. For this, l-Ala transport was measured in parasites previously incubated for 30 min with 5 µg/ml oligomycin A or not (with a control, where the oligomycin is added without preincubation, to assess if this ATP synthase inhibitor does not have an off-target effect on the transport system), as it was previously demonstrated that this 30-min treatment decreases the intracellular ATP levels by 60%, while without preincubation, it did not affect the ATP levels (29, 30). The fact that we observed a significant diminution of l-Ala uptake (48.6% ± 8.8%) only in parasites preincubated for 30 min with oligomycin A indicates that this is an active process (Table 2). In addition, l-Ala uptake was decreased in the presence of carbonyl cyanide *p*-trifluoromethoxyphenylhydrazone (FCCP) alone (44.2% ± 11.1%), which supports the possibility of transport being dependent on an H^+^ gradient across the plasma membrane as the driving force (31). However, it is known that FCCP, as an uncoupler, collapses the mitochondrial inner membrane potential (ΔΨm), and as a response to this, the F_1_F_o_ ATP-synthase could start to work in “reverse mode” (32), hydrolyzing ATP to pump protons to the intermembrane mitochondrial space. Thus, FCCP treatment triggers in fact at least two simultaneous effects: (i) the collapse of transmembrane proton gradients and (ii) the depletion of the intracellular ATP pools. To separate both effects, we measured the l-Ala uptake in cells simultaneously treated with FCCP and oligomycin A (without preincubation). The combined treatment impaired the l-Ala transport (53.4% ± 9.9%) (Table 2). In addition, l-Ala uptake was also assayed in the presence of carbonyl cyanide *m*-chlorophenylhydrazone (CCCP) at various concentrations (0, 10, 20, and 100 µM) to verify whether l-Ala can be incorporated in a transmembrane proton gradient-independent manner (see Fig. S2 in the supplemental material). Our data showed that 40% of l-Ala is incorporated at a high concentration of H^+^ gradient uncoupler. Taken together, these results indicate that l-Ala transport system is partially dependent on a transmembrane proton gradient as the driving force, which is maintained by plasma membrane P-type H^+^-ATPases (31).

10.1128/mSphereDirect.00338-18.3 FIG S2  Effect of different CCCP concentrations on l-Ala transport in epimastigotes of Trypanosoma cruzi. The assay was performed as described in Materials and Methods for the results shown in Table 2. Download FIG S2, TIF file, 0.1 MB. Copyright © 2018 Girard et al. 2018 Girard et al. This content is distributed under the terms of the Creative Commons Attribution 4.0 International license.

**TABLE 2:** Effect of oligomycin A and FCCP on l-Ala uptake in Trypanosoma cruzi epimastigotes

Addition(s)	% of l-Ala uptake inhibition
Control	0
Oligomycin A (5 µg/ml)	
0 min	4.2 ± 7.9
30 min	48.6 ± 8.8
FCCP (0.5 µM)	44.2 ± 11
Oligomycin A (5 µg/ml) + FCCP (0.5 µM)	53.4 ± 9.9

### The bioenergetics of T. cruzi with l-Ala as the substrate.

**FIG 2:**
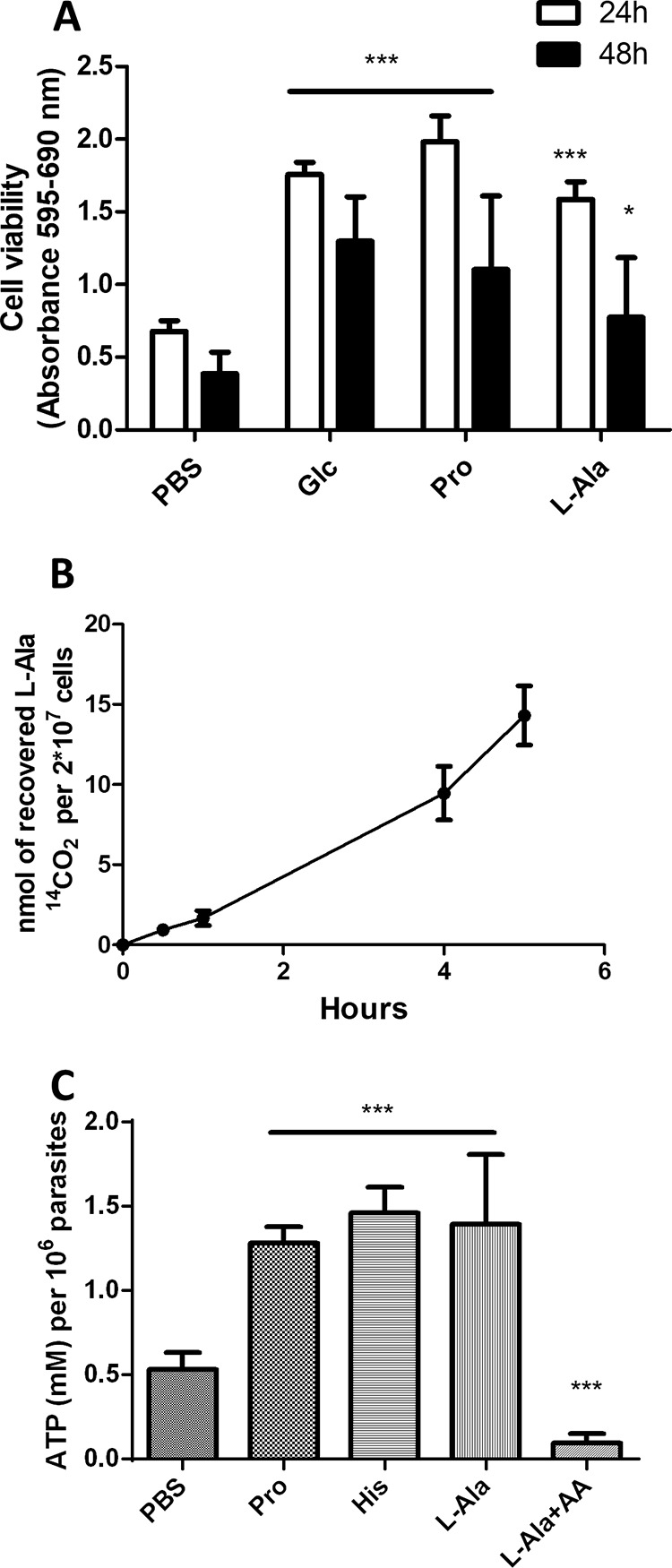
The effect of l-Ala on bioenergetics parameters. (A) Viability assays of epimastigotes of T. cruzi using l-Ala as the only energy source. An MTT assay was used to assess epimastigotes in the exponential phase of growth (LIT medium) transferred to supplemented PBS. The viability was expressed as a percentage of formazan formation through the reduction of MTT with respect to the maximum viability obtained for this assay when cells were treated with Glc (glucose) or Pro (proline), both taken as positive controls. The parasites growing initially in LIT medium were transferred to supplemented PBS (or not [negative control]) with different substrates, including Glc and Pro as positive controls and were incubated for 24 or 48 h. Here, we consider the positive controls as viable parasite populations. (B) ^14^CO_2_ production from epimastigotes incubated in 5 mM l-[^14^C]Ala after 30, 60, 240, and 300 min. (C) ATP production from l-Ala catabolism. The intracellular ATP content after 60 min of recovery in parasites nutritionally stressed using the substrates indicated is shown. The ATP concentration was determined using a luciferase assay, and the data were normalized by the total cell number. AA, antimycin A (0.5 µM). One-way analysis of variance (ANOVA) followed by a Tukey’s posttest was used for statistical analysis to compare the values to those from the respective control. ***, *P* < 0.001; *, *P* < 0.05 (Tukey’s posttest). Panels A, B, and C correspond to mean values from three independent biological experiments.

To evaluate the role of l-Ala in the energy metabolism of T. cruzi, different bioenergetics parameters were evaluated. Initially, the viability of the parasites when incubated in PBS supplemented with this amino acid as the only energy source was evaluated and compared to the survival measured by supplementing PBS with other energy sources or not (controls). Thus, exponentially growing epimastigotes were incubated in PBS (negative control) or PBS supplemented with 5 mM l-Ala, 5 mM Pro, or 5 mM glucose. (The latter two are known energy sources for T. cruzi epimastigotes and therefore were used as positive controls.) After 24 and 48 h, cell viability was measured. The parasites incubated in l-Ala had their viability increased compared to those in nonsupplemented PBS (Fig. 2A) at both incubation times, suggesting that l-Ala can be metabolized as an energy source. In fact, once taken up by the cells, l-Ala can be converted into pyruvate by an Ala dehydrogenase, which is essentially glycosomal (25), or a transaminase accepting Ala as a substrate, such as the Tyr or Ala transaminases, which can be both mitochondrial and cytosolic (33). Then, pyruvate can be converted into malate by the cytosolic malic enzyme (34–37), and subsequently into fumarate or succinate, in the mitochondrion or glycosomes (38). Alternatively, pyruvate can be converted into acetyl coenzyme A (acetyl-CoA) through the pyruvate dehydrogenase complex (see Fig. 5) (20, 25). Whatever the case, at least part of the pyruvate may have as a final destiny its total oxidation to CO_2_ plus H_2_O (39). In order to verify this possibility, epimastigotes were incubated for 30, 60, 240, and 300 min with l-[U-^14^C]Ala, and the time-dependent ^14^CO_2_ production was monitored (Fig. 2B). After a 60-min incubation, we observed that 10% of the transported amino acid was oxidized to CO_2_, while 65% of the detected radioactivity was incorporated into soluble metabolites, corresponding to free l-Ala and to the l-Ala eventually incorporated into other soluble macromolecules, and approximately 25% was incorporated into trichloroacetic acid-precipitable macromolecules (Table 3).

**TABLE 3:** l-Ala incorporated in the soluble and insoluble fractions and ^14^CO_2_ produced from epimastigotes incubated in 5 mM l-[U-^14^C]Ala for 1 h

Fraction or CO_2_	l-[^14^C]Ala incorporation or ^14^CO_2_ production (nmol/1 × 10^7^ cells)	% of l-Ala incorporation or ^14^CO_2_ production
Pellet	2.6 ± 0.39	24.7
Supernatant	6.9 ± 1.3	65.3
CO_2_	1.1 ± 0.5	10

**FIG 3:**
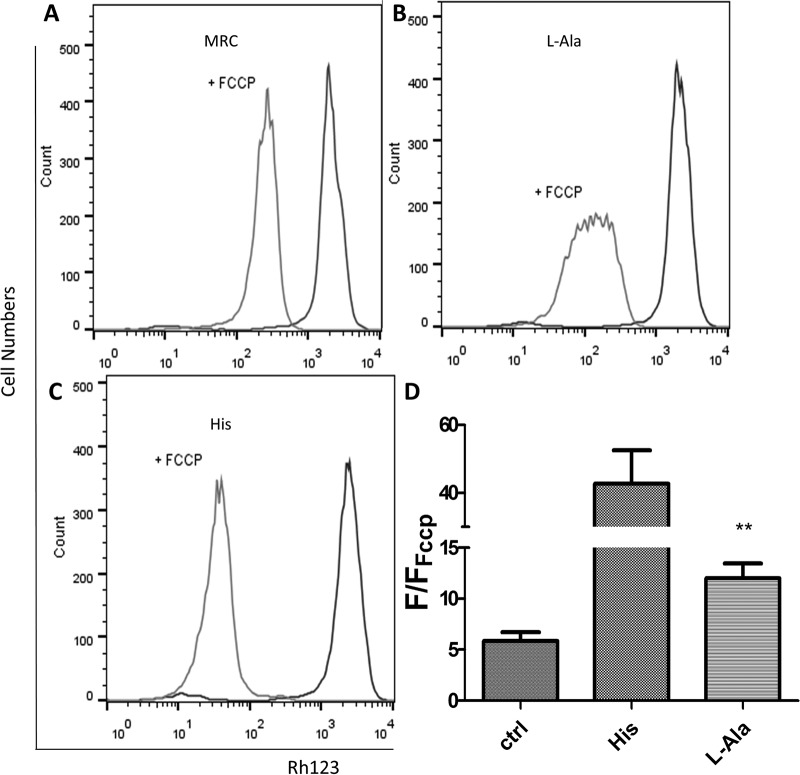
Mitochondrial inner membrane potential by l-Ala catabolism. Flow cytometry analysis shows the fluorescence in epimastigotes incubated with rhodamine 123 after 30 h of nutritional stress and recovery or not (A) with l-Ala (B) or (C) His (C). “+ FCCP” indicates the fluorescence shift after the addition of the uncoupling agent. (D) The fluorescence ratios between the coupled and uncoupled parasites under each condition were calculated using the geometric mean (area under each peak). Samples were compared to the control using the *t* test. **, *P* < 0.01. The data correspond to four independent biological experiments.

**FIG 4:**
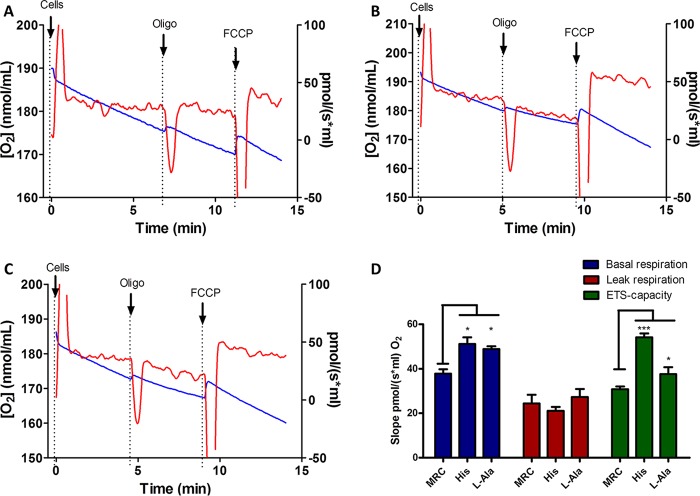
Respiratory rates in recovered epimastigotes. Oxygen consumption was measured after starvation (16 h in PBS) without recovery (A) as a negative control and recovered by adding 5 mM His (B) for 30 min as a positive control or 5 mM l-Ala (C) for 30 min. The red lines indicate the variation in oxygen concentration as a function of time (right axis). The blue lines represent O_2_ concentration (left axis). Oligo, oligomycin A at 0.5 µg/ml. FCCP was used at 0.5 µM. (C) Basal respiration (initial oxygen flux values), leak respiration after the addition of 0.5 µg/ml of oligomycin A, and electron transfer system (ETS) capacity after the addition of 0.5 µM FCCP were measured for each condition. One-way ANOVA followed by a Tukey’s posttest was used for statistical analysis to compare the values to the respective control. ***, *P* < 0.001; *, *P* < 0.05 (Tukey’s posttest). The data correspond to three independent biological experiments.

The ability of l-Ala to support ATP biosynthesis was also evaluated. Epimastigotes were subjected to a severe metabolic stress by incubating them for 30 h in PBS in the absence of any energy source. The cells were then evaluated for recovery of their ATP levels by incubating them for 1 h in the presence of 5 mM l-Ala, 5 mM Pro, or 5 mM His. Pro and His were positive controls since it was previously demonstrated that both are able to recover the intracellular ATP levels diminished by starvation (7, 40). A negative control consisting of keeping the parasites for 1 h in the absence of any metabolite was also performed. Then, intracellular ATP was measured by a luciferase assay. Parasites incubated in the presence of l-Ala showed a significant increase in the intracellular amounts of ATP compared to those in the negative control, and this increase was in the range of those obtained with Pro and His. Interestingly, the recovery of ATP levels was abolished by the addition of antimycin A (0.5 µM), a respiratory chain (complex III) inhibitor. These data strongly suggest that l-Ala can be used for fueling ATP synthesis through oxidative phosphorylation (Fig. 2C; see Fig. S3 in the supplemental material). As our results showed that l-Ala has a role in T. cruzi bioenergetics, we evaluated whether this amino acid is involved in maintaining the mitochondrial inner membrane potential (ΔΨm). The cells were preincubated for 30 h in PBS buffer to induce a severe metabolic stress and then recovered by incubation for 1 h in 5 mM l-Ala in mitochondrial cellular respiration (MCR) buffer, 5 mM His in MCR buffer (positive control), or MCR buffer alone (negative control). Then, rhodamine 123, a fluorescent ΔΨm indicator was added, and the fluorescence measurements were made by cytometry (7). The degree of recovery of ΔΨm was calculated on the basis of the ratio of the fluorescence by cells not treated with FCCP (polarized mitochondrial membrane) and FCCP-treated (depolarized mitochondrial membrane) cells under each condition. The l-Ala-recovered cells restored their ΔΨm compared to the negative control, although the ability of this amino acid to restore ΔΨm was significantly lower than that of His (Fig. 3C). As l-Ala was able to maintain the parasites’ viability and sustain ATP production and the ΔΨm, we also measured its ability to trigger parasite respiration. Epimastigotes were incubated for 16 h and then recovered with different substrates or not (basal respiration) for 30 min. His was used as positive control (7). The rates of O_2_ consumption were measured after the addition of cells, stimulated or not with l-Ala (basal respiration), then inhibited by the addition of oligomycin A, and finally uncoupled by FCCP to determine the leak of respiration and the maximum capacity of the electron transport system (ETS), respectively (Fig. 4). Our results demonstrate that, after 30 min of incubation, l-Ala triggered an O_2_ consumption level that is similar to that recorded with His and higher than that of the nonstimulated parasites (Fig. 4). As expected, respiration rates triggered by l-Ala were inhibited by oligomycin A and then stimulated by FCCP, demonstrating its oxidation through the respiratory chain (Fig. 4D). Summarizing, our results show that l-Ala can deliver electrons to the respiratory chain and fuel ATP synthesis through oxidative phosphorylation in epimastigotes.

10.1128/mSphereDirect.00338-18.4 FIG S3  ATP production from l-Ala catabolism. The intracellular ATP content after 60 min of recovery in parasites nutritionally stressed using the substrates indicated is shown. The ATP concentration was determined using a luciferase assay, and the data were normalized by the total cell number. AA, antimycin A (0.5 µM). One-way ANOVA followed by Tukey’s posttest was used for statistical analysis to compare the values to the nonrecovered parasites. ***, *P* < 0.001; **, *P* < 0.01 (Tukey’s posttest). The data represent the three biological assays (A, B, and C). Download FIG S3, TIF file, 0.2 MB. Copyright © 2018 Girard et al. 2018 Girard et al. This content is distributed under the terms of the Creative Commons Attribution 4.0 International license.

## DISCUSSION

Ala is available throughout T. cruzi’s life cycle. In the insect vector, this amino acid is present in both the hemolymph and the excreta (41–43). Ala is also available to the parasite forms when residing within the mammalian cells and in the plasma (44). Additionally, as mentioned, the parasite produces two independent pools of Ala as a consequence of its own metabolism (17, 18, 28, 33). These facts raise the importance of studying in more detail its uptake and subsequent metabolism.

Most of the biochemically characterized amino acid transport systems in T. cruzi showed functional characteristics that are compatible with those of members of the AAAP (amino acid/auxin permease) family, a family grouping H^+^/amino acids and auxin permeases (45). Noteworthy, for T. cruzi the uptake of most of amino acids has already been biochemically analyzed (2).

In the present work, we biochemically described the uptake of l-Ala by T. cruzi epimastigotes. We identified a single transport system with a *K*_m_ similar to values already described for branched-chain amino acids (BCAAs), γ-aminobutyric acid (GABA), Glu, and Pro by transport system A. However, the *V*_max_ value is, to our knowledge, the highest reported until now for any amino acid transport system in T. cruzi (2). These data could in part explain the reported rapid changes in the concentration of intracellular Ala in cells under hyper- or hypo-osmotic stress (26–28). Such a function in osmoregulation was also attributed to the l-Ala transporters in *Leishmania* spp. (46–48).

**FIG 5:**
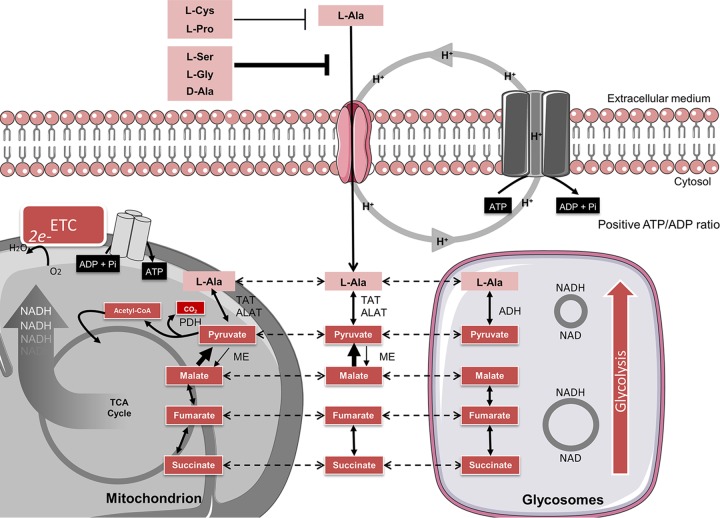
Schematic proposal for the uptake and catabolism of l-Ala in T. cruzi. The glycosomal and mitochondrial compartments and the TCA cycle are indicated. The metabolic flux at each enzymatic step is represented by arrows of different thicknesses. Dashed arrows indicate the intracellular shuttle of the molecules between different compartments. ETC, electron transport chain; TAT, tyrosine aminotransferase; ALAT, Ala aminotransferase; ADH, Ala dehydrogenase; ME, malic enzyme; PDH, pyruvate dehydrogenase.

Regarding the specificity, our results showed that amino acids structurally related to l-Ala, like short-chain amino acids (Ser and Gly), as well as neutral amino acids (Pro and Cys), compete with l-Ala for uptake. The transport activity increased exponentially at temperatures between 15 and 40°C, a range to which the parasites could be naturally exposed inside the insect vectors (49). Given this, we may assume that the environmental temperature is a natural modulator of l-Ala uptake. The obtained *E*_*a*_ was in the range of those reported for other amino acid transport systems, with the low-afﬁnity Arg transporter as the only exception (30). Interestingly, the *E*_*a*_ corresponds approximately to a requirement for the hydrolysis of 2 molecules of ATP into ADP plus P_i_ per l-Ala molecule transported into the cells. Additionally, our data showed that l-Ala uptake is mediated by an active process and occurs similarly to that of l-Ala uptake in *Leishmania* and the transport of most amino acids in T. cruzi (2). The main driving force in these processes is a transmembrane H^+^ gradient, most likely created by a plasma membrane-located proton-pumping ATPase (Table 2; Fig. 5) (31).

As mentioned before, it has been well described that the T. cruzi epimastigotes’ proliferation is initially based on the consumption of glucose (when available) and then, after the exhaustion of this metabolite, on the consumption of available amino acids (13, 17). As a consequence of the glucose consumption, the cells mainly produce succinate acetate and CO_2_ (50–52), while significant amounts of NH_4_^+^ are produced when glucose is scarce (50, 53, 54), derived from the increased consumption of amino acids (7, 9, 13, 55). In T. cruzi, at least two systems have been proposed as being involved in the detoxification of the metabolically produced NH_4_^+^ (56, 57). At the same time, NH_4_^+^ can be used for NADH oxidation. This happens through the concerted action of (i) Glu dehydrogenases (which use NH_4_^+^ to aminate α-ketoglutarate, yielding Glu) and (ii) Ala, Asp, and Tyr transaminases, all of which have Glu and pyruvate as cosubstrates and are able to catalyze the transfer of the −NH_2_ group from Glu into pyruvate, yielding Ala and regenerating the α-ketoglutarate, as previously proposed (2, 56). Thus, Ala is a major end product of the combined glucose and amino acid metabolism. However, some data in the literature (10, 25, 58) suggest also the possible catabolism of l-Ala.

Our results indicate that l-Ala can be a “fuel for life” instead of being merely a catabolic end product to be secreted. Indeed, the presence of all enzyme activities and complexes that would be critical for enabling the complete oxidation of this amino acid to support ATP production by oxidative phosphorylation have been demonstrated: (i) l-Ala can be converted into pyruvate by different transaminases (14, 21–23, 59–61), (ii) pyruvate can be converted into acetyl-CoA and further oxidized through the tricarboxylic acid (TCA) cycle (17, 20, 25), and (iii) pyruvate can be converted into malate by the cytosolic malic enzyme—even taking into account that the reaction in the pyruvate→malate direction is very slow (36)—and further into fumarate and succinate in two subcellular compartments, the mitochondrion or the glycosomes, the peroxisome-related organelles of trypanosomatids (17, 38, 39, 62–64). All these metabolites can be used as intermediates or as fuel for the TCA cycle, thus allowing (in principle) their full oxidation (20, 25) (Fig. 5) to feed the energy metabolism. Together, our results demonstrate that extracellular l-Ala is at least partially catabolized to CO_2_ and used for ATP production by oxidative phosphorylation. Despite the differences reported by several authors about the metabolism of this amino acid between T. cruzi and *Leishmania* spp. (2, 17, 38), it is tempting to stress that our results are consistent with those previously obtained with Leishmania major and Leishmania braziliensis (65–67), which also point to a complete l-Ala oxidation. Notably, the plasticity of l-Ala metabolism underlines the relevance of T. cruzi’s metabolic flexibility to adapt to different environmental conditions.

In conclusion, l-Ala can be produced and secreted as a main end product of the metabolism of glucose and amino acids by T. cruzi epimastigotes, while in the absence of glucose and at high concentrations, it can be taken up by the cells and further oxidized with production of CO_2_, triggering O_2_ consumption, contributing to the maintenance of the inner mitochondrial membrane potential and powering ATP production through oxidative phosphorylation.

## MATERIALS AND METHODS

### Reagents.

l-[U-^14^C]Ala (0.1 mCi/ml) was purchased from American Radiolabeled Chemicals, Inc. (ARC [St. Louis, MO]). All other reagents were from Sigma (St. Louis, MO).

### Parasites.

T. cruzi CL strain clone 14 epimastigotes (68) were maintained in the exponential growth phase by subculture every 48 h in liver infusion tryptose (LIT). Medium supplemented with 10% fetal calf serum (FCS) at 28°C. For transport assays, exponentially growing parasites were washed three times with PBS (NaCl, 137 mM; KCl, 2.6824 mM; Na_2_HPO_4_, 8 mM; and KH_2_PO_4_, 1.4694 mM, pH 7.2) and resuspended to a final density of 2 × 10^8^ cells/ml in PBS. To evaluate the ability of epimastigotes to use l-Ala to resist a severe metabolic stress and as an energy source, parasites in the exponential growth phase (5 × 10^7^ parasites per ml obtained from a 24-h culture started at 2.5 × 10^7^ parasites per ml) were washed twice in 1 volume of PBS and incubated for 30 h in 1 volume of the same buffer. After incubation, l-Ala was added to the cultures at a saturating concentration (5 mM) for its uptake, and different parameters of energy metabolism were determined, including cell viability, ATP production, oxygen consumption, and mitochondrial inner membrane potential. In all cases, the viability of the parasites was evaluated by microscopic observation of cell motility.

### Transport assays.

Transport assays were performed as described previously (40). Transport assays were initiated by the addition of 100 µl of 5 mM l-Ala in PBS to aliquots of parasites of 100 µl (2 × 10^7^ cells each, except when otherwise specified, traced with 0.4 µCi of l-[U-^14^C]Ala). The uptake was measured at 28°C for 1 min, except when otherwise specified. The transport reaction was stopped by addition of 800 µl of stop solution (50 mM l-Ala in PBS, pH 7.4) prechilled at 4°C, immediately followed by two washes with cold PBS. Background values in each experiment were measured by the simultaneous addition of each traced amino acid and stop solution (29).

### Competition assays.

Competition assays were performed by measuring l-Ala uptake at a concentration equivalent to the *K*_m_ in the presence of 10 times excess of each other amino acid (29). Briefly, 100-µl aliquots of parasites containing 2 × 10^7^ cells were incubated with the transport solution supplemented with the presumably competing metabolite for 1 min. The results obtained were expressed as inhibition percentages in relation to a control (the same experiment without the competitor).

### The effect of extracellular ions, pH, and energy.

The incorporation of l-Ala in the presence of Na^+^ and K^+^ was measured by comparing the l-Ala uptake using a conventional PBS with the same composition as described previously, a Na^+^-free PBS in which all Na^+^ was replaced by K^+^ (149.5 mM KCl [called here Na^+^-free PBS]), a K^+^-free PBS in which all K^+^ was replaced by Na^+^ (149.5 mM Na^+^ [called here K^+^-free PBS]), and a phosphate buffer in which the ionic strength was supplied by choline (149.5 mM choline [called here PBS-choline]) as the control. The effect of extracellular pH was determined by measuring the transport using buffers with different pHs. According to their buffer capacity, experiments were performed using PBS for the pH range between 6.0 and 7.5, and citrate was used for the pH range between 5.0 and 6.5.

The effect of a proton-dependent plasma membrane potential on the l-Ala uptake in parasites treated with 0.5 µM the protonophore carbonyl cyanide *p*-trifluoromethoxyphenylhydrazone (FCCP) was evaluated. As previously reported, FCCP treatment can affect an uptake process due to the disruption of the H^+^ gradient across cellular membranes (if the uptake is performed through a H^+^/metabolite symporter) or to the diminution of intracellular levels of ATP due to its rapid consumption by the mitochondrial F_1_F_o_-ATP synthase, which in a low-mitochondrial-membrane-potential situation hydrolyzes ATP to pump H^+^ to reestablish the mitochondrial inner membrane potential (40). To discriminate between both effects, a control was performed with the addition of 5 µg/ml oligomycin A to FCCP-treated cells, which allowed simultaneous disruption of H^+^ membrane gradients while blocking the F_1_F_o_-ATPase.

The viability of the parasites under all conditions was verified by observing their motility under the microscope.

### Analysis of data.

The disintegrations per minute (dpm) corresponding to transported radiolabeled l-Ala for each experimental point (dpm_*i*_) were calculated as dpm_*i*_ = dpm_*e*_ − dpm_*b*_, where dpm_*e*_ is the average dpm from triplicates after 1 min of incubation in the presence of radiolabeled l-Ala and dpm_*b*_ is the average dpm from the background samples.

l-Ala taken up by the cells was calculated as l-Ala_*i*_ = dpm_*i*_ [l-Ala] *v* dpm*_st_*^−1^ *t*^−1^, where l-Ala_i_ is the transported l-Ala, [l-Ala] is the l-Ala nanomolar concentration, *v* is the volume of radiolabeled l-Ala, dpm_*st*_ is the total dpm measured for each added radiolabeled l-Ala, and *t* is the time of incubation measured in minutes.

### Statistical analysis.

Curve adjustments, regressions, and statistical analysis were performed with the GraphPad Prism 5 analysis tools. All assays were performed at least in biological triplicate, and the details of statistical analysis were added to each figure legend.

### Estimation of number of transporters by using the Arrhenius equation.

From a thermodynamic point of view, a transporter is nothing other than a type of enzyme catalyzing, in this case, the reaction l-Ala_*e*_ = >l-Ala_*i*_, where l-Ala_*i*_ is the intracellular l-Ala and l-Ala_*e*_ is the extracellular l-Ala.

To estimate a number of transport systems, it is necessary to measure the turnover of active sites (*k*_cat_). In turn, *k*_cat_ = *V*_max_/no. of transporters (69).

For these systems, we proceeded to set up as a hypothesis to be tested that, at the saturated substrate concentration, the system is limited by the dissociation step. Then we can estimate the number of transporter sites at defined temperature using the Arrhenius equation as follows:

$$\text{no. of transporters}\;=\;\frac{V_{\max}}{{Ae}^{-\frac{E_{a}}{RT}}}$$

where *V*_max_ is the maximum rate achieved by the system at saturating substrate concentration at a given temperature, *Ae* is the pre-exponential factor, *E*_*a*_ is the activation energy for the reaction, *R* is the universal gas constant, and *T* is the temperature of the reaction (in kelvins).

### The contribution of l-Ala to recover cells subjected to a severe metabolic stress.

To determine whether l-Ala is able to restore the viability of epimastigotes of T. cruzi after a starvation period, the parasites were exponentially cultured in LIT and stressed in PBS as described above. Briefly, the epimastigotes (5 × 10^7^ cells) were incubated for 24 and 48 h at 28°C in PBS plus 5 mM l-Ala to induce cell recovery. Separate treatments in glucose or proline were used as controls. After recovery, the cells were washed in PBS and incubated with 3-(4,5-dimethyl-2-thiazolyl)-2,5-diphenyl-2H-tetrazolium bromide (MTT) reagent to evaluate cell viability, as previously described (7).

### Mitochondrial inner membrane potential determination.

To assess the ability of l-Ala to energize the mitochondria, parasites (5 × 10^7^ cells per ml) were starved as described above. The stressed parasites were then incubated for recovery in MCR buffer (125 mM sucrose, 65 mM KCl, 10 mM HEPES-NaOH, pH 7.2, 1 mM MgCl_2_, 2 mM K_2_HPO_4_) supplemented with 5 mM l-Ala or 5 mM His (positive control). Nonsupplemented MCR buffer treatment was used as negative control. Parasites were incubated with 250 nM rhodamine 123 (Sigma) for 20 min at 28°C, washed with cytomix buffer (25 mM HEPES-KOH, 120 mM KCl, 0.15 mM CaCl_2_, 2 mM EDTA, 5 mM MgCl_2_, 10 mM K^+^-phosphate buffer, pH 7.2, and 10 µM FCCP if required). Changes in the fluorescence of cells labeled with rhodamine 123 were analyzed by flow cytometry. Parasites were analyzed in an FL-1 detector of a FACSCalibur flow cytometer using CellQuest Pro software (Becton, Dickinson, NJ, USA). The relative change in ΔΨm was determined as the ratio between both conditions (the coupled and uncoupled states elicited by FCCP).

### ATP biosynthesis dependency of l-Ala.

To evaluate ATP production with l-Ala as their sole energy source, the parasites (approximately 5 × 10^7^ cells per ml) were starved as described above and recovered or not (negative control) by incubation for 1 h in the presence of 5 mM His or Pro (as positive controls) or 5 mM l-Ala. The intracellular concentration of ATP in each sample was determined before and after recovery by using a luciferase assay according to the manufacturer’s instructions (Sigma). ATP concentrations were estimated by using a calibration curve (ATP disodium salt, Sigma); luminescence (λ570 nm) was detected using a SpectraMax i3 plate reader (Molecular Devices, Sunnyvale, CA).

### Incorporation of l-Ala into proteins.

To estimate the percentage of labeled l-Ala that was incorporated into proteins, the cells were incubated for 60 min with 5 mM l-Ala in the presence of 0.1 µCi of l-[U-^14^C]Ala. The parasites were washed twice and resuspended in 500 µl of PBS. Then the cells were treated with 1 volume of 20% trichloroacetic acid, incubated for 1 h at room temperature, and centrifuged for 30 min at 10,000 × *g*. The pellets were resuspended in 0.1% SDS in a 15 mM Tris-HCl buffer (pH 7.4). The supernatants and pellets were resuspended in a scintillation cocktail. The amount of radioactivity incorporated into the macromolecules was measured by a scintillation counter (PerkinElmer Tri-Carb 2910TR).

### CO_2_ production measurements.

To measure the CO_2_ production from the tricarboxylic acid (TCA) cycle during l-Ala catabolism, epimastigotes exponentially growing in LIT (5 × 10^7^ parasites per ml) were washed twice, resuspended in PBS and incubated in 5 mM l-Ala spiked with 0.1 µCi of l-[U-^14^C]Ala for 0.5, 1, 4, and 5 h at 28°C. To trap the produced CO_2_, pieces of Whatman filter embedded in 2 M KOH were placed on the top of the tubes in which the parasites were incubated. The filters were recovered and mixed with scintillation cocktail, and the K_2_^14^CO_3_ production on the paper was measured by using a scintillation counter.

### Oxygen consumption.

The rates of oxygen consumption were measured using intact cells in a high-resolution oxygraph (Oxygraph-2k; Oroboros Instruments, Innsbruck, Austria). To evaluate the O_2_ consumption rates from l-Ala, the exponentially growing parasites (5 × 10^7^ cells per ml) were washed twice in PBS, subjected to nutritional stress for 16 h in the same buffer, and recovered with 5 mM l-Ala or His (positive control) for 30 min at 28°C. The parasites were added to the MCR buffer. Oligomycin A (0.5 µg/ml) and FCCP (0.5 µM) were sequentially added to measure the optimal noncoupled respiration and leak state of respiration, respectively. Data were recorded and treated by using DatLab 7 software.
