# Correction for Girard et al., “Uptake of l-Alanine and Its Distinct Roles in the Bioenergetics of *Trypanosoma cruzi*”

**DOI:** 10.1128/mSphere.00657-18

**Published:** 2018-12-12

**Authors:** Richard M. B. M. Girard, Marcell Crispim, Mayke Bezerra Alencar, Ariel Mariano Silber

**Affiliations:** a Department of Parasitology, Laboratory of Biochemistry of Tryps - LaBTryps, Institute of Biomedical Sciences, University of São Paulo, Cidade Universitária, Butantã, São Paulo, Brazil

## AUTHOR CORRECTION

Volume 3, no. 4, e00338-18, 2018, https://doi.org/10.1128/mSphereDirect.00338-18. The following is not a correction *per se* but rather a clarification of some points in our published article. We calculated the kinetic and thermodynamic properties of the alanine uptake system in Trypanosoma cruzi using the Arrhenius equation (ln *V*_0_ = ln *A* × *E_a_*/RT). We know that the following version of the Arrhenius equation can be used to calculate the thermodynamic properties of this process: ln *k* = ln *A* × *E_a_*/RT, where *k* = *V*_0_/*n* (*n* = number of transporters). We want to clarify that we used the former equation because under the conditions of our experiments we assumed that the number of transporters present in the cell would be constant and unaffected by denaturation, cell lysis, or other effects. We confirmed the validity of this assumption by determining that, in the range of temperatures used for the determination of *E_a_*, ln *V*_0_ versus 1/*T* fits well to a linear function since variation would be indicated by a variation in *n*. We would like to remark as well that, except otherwise indicated, the graphs correspond to a representative experiment in triplicates of at least three independent replicas.

