# Supplementary figures and images for: Uptake of l-Alanine and Its Distinct Roles in the Bioenergetics of Trypanosoma cruzi
Source: mSphere. 2018 Jul 18;3(4):e00338-18. doi: 10.1128/mSphereDirect.00338-18 (PMC6052336; doi:10.1128/mSphereDirect.00338-18)

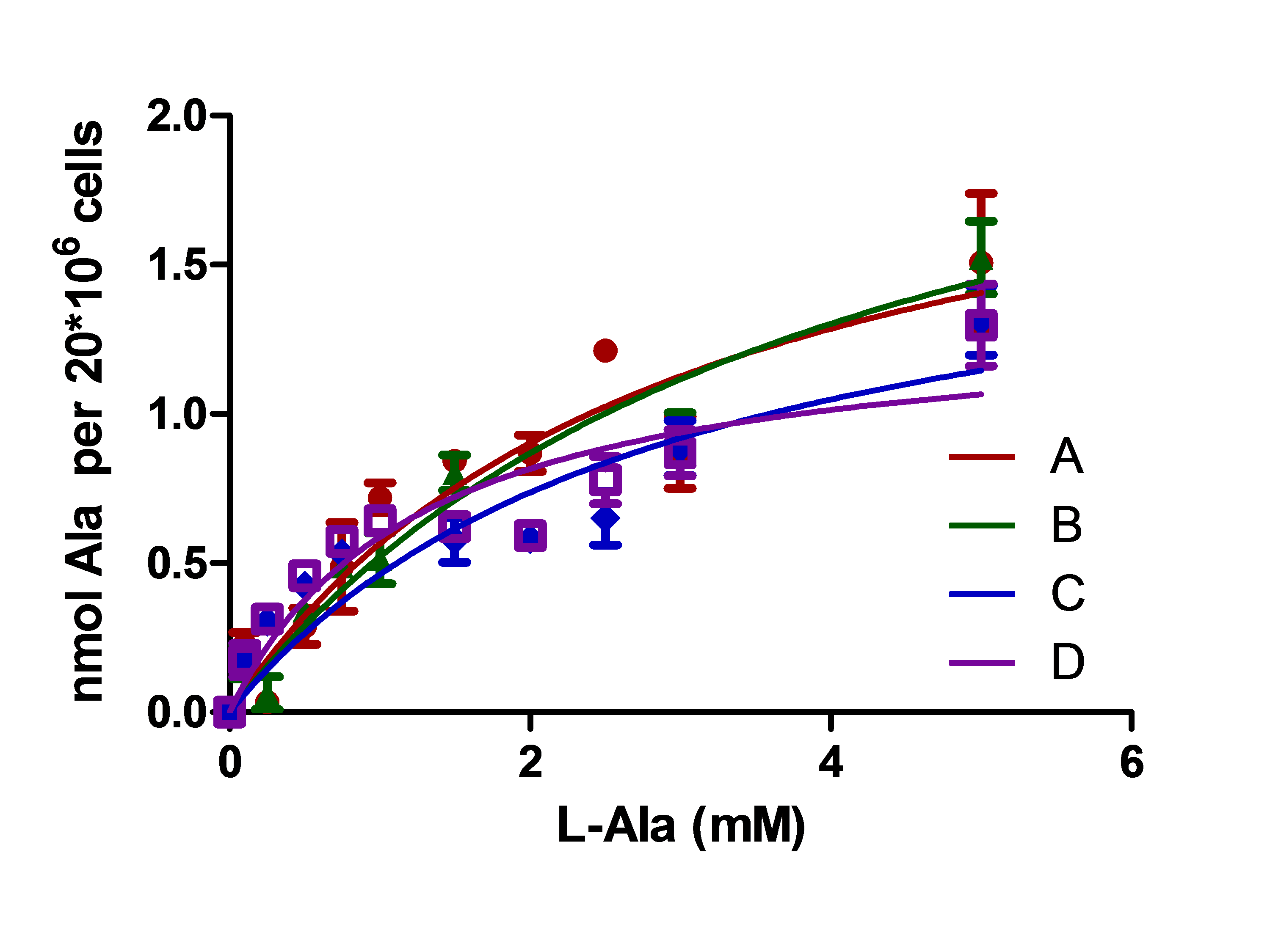

Supplement: FIG S1 [file sph004182594sf1.tif]

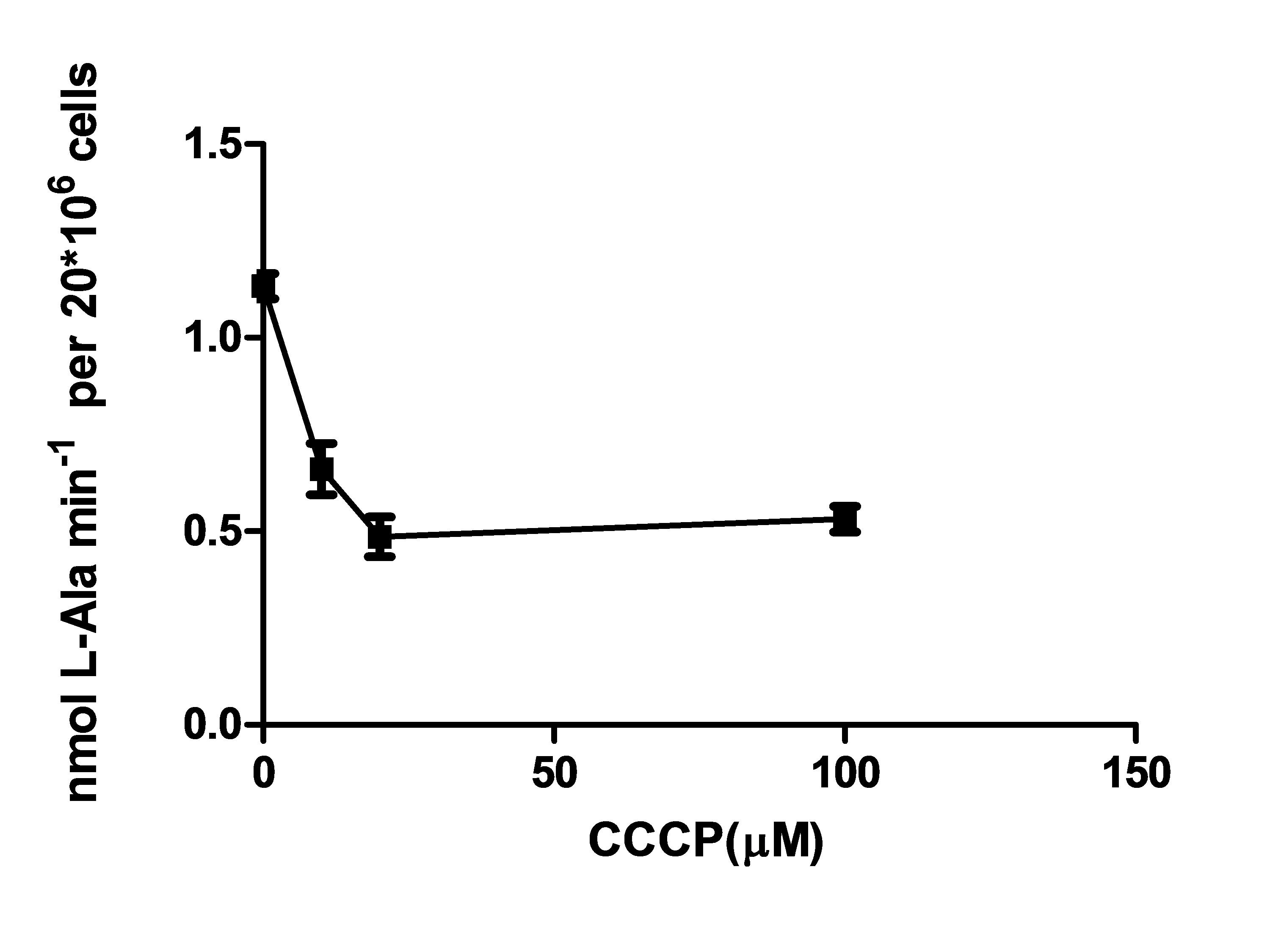

Supplement: FIG S2 [file sph004182594sf2.tif]

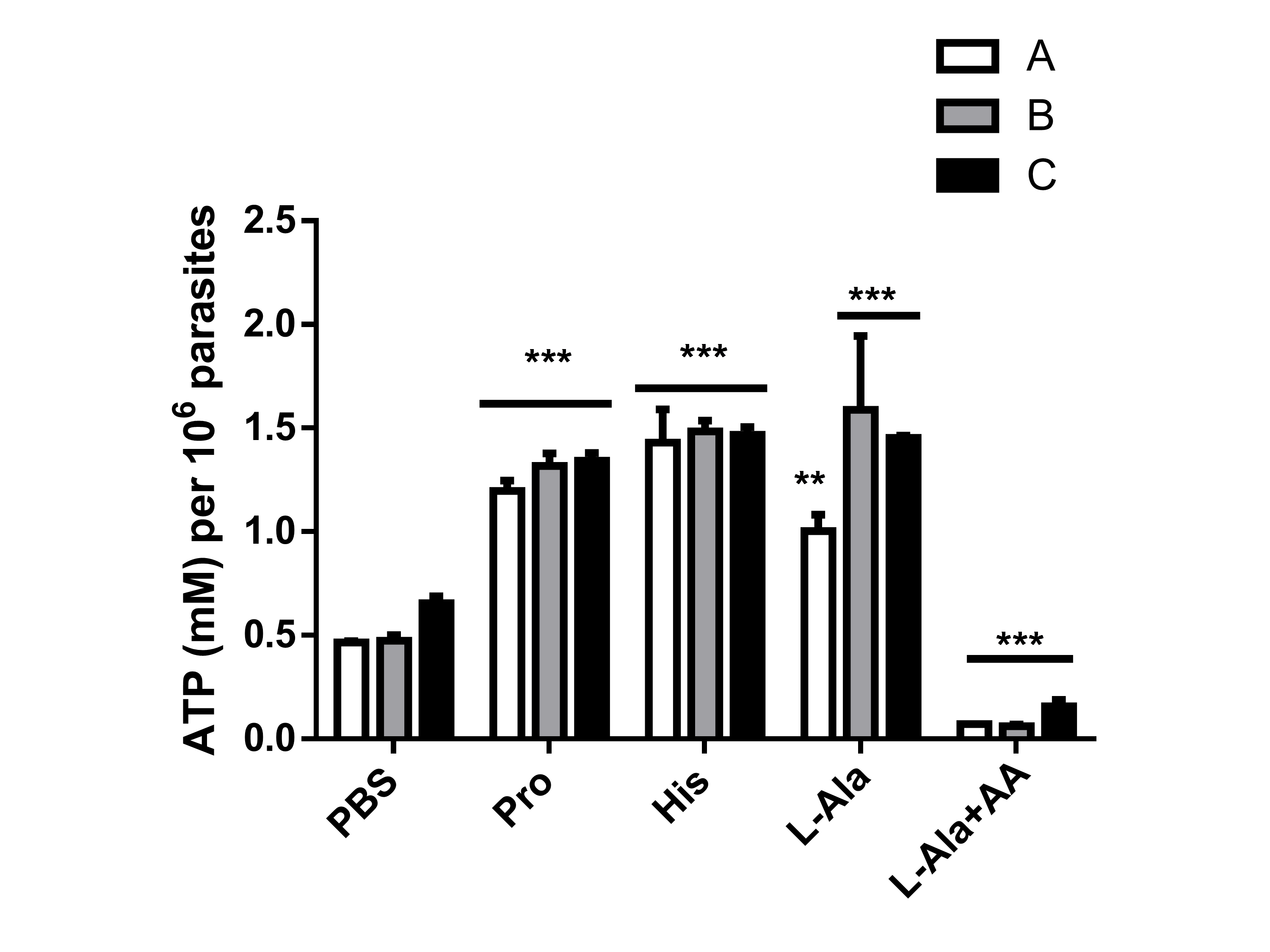

Supplement: FIG S3 [file sph004182594sf3.tif]
