# Supplementary material for: Uptake of l-Alanine and Its Distinct Roles in the Bioenergetics of Trypanosoma cruzi
Source: mSphere. 2018 Jul 18;3(4):e00338-18. doi: 10.1128/mSphereDirect.00338-18 (PMC6052336; doi:10.1128/mSphereDirect.00338-18)
Supplement: TABLE S1 [file sph004182594st1.docx]

| **Michaelis-Menten best fits:** | **A** | **B** | **C** | **D** | **Average** | **SD** |
| --- | --- | --- | --- | --- | --- | --- |
| ***V*_max_** | 1.72 | 2.24 | 2.06 | 1.44 | 1.86 | 0.31 |
| ***K*_M_** | 0.43 | 2.96 | 2.38 | 1.49 | 1.81 | 0.61 |

**Table S1**
