# Supplementary material for: Uptake of l-Alanine and Its Distinct Roles in the Bioenergetics of Trypanosoma cruzi
Source: mSphere. 2018 Jul 18;3(4):e00338-18. doi: 10.1128/mSphereDirect.00338-18 (PMC6052336; doi:10.1128/mSphereDirect.00338-18)
Supplement: TEXT S1 [file sph004182594s1.docx]

Computation:

Kcat = A*exp(-Ea/R*T)

At 28°C, [S] at saturate concentration

R has the value of 8.314 x 10-3 kJ mol^-1^K^-1^

A = 9.7x10^9^ (from the Arrhenius Equation plot)

Ea = 66.4 kj/mol

75.4 ++ => kcat 0.00080898 **min^-1^  = 0.48 s^-1^**

57.4-- => kcat 1.023 **min^-1^ = 61.38 s^-1^**

T = 301.15 K

Kcat = A*exp(-Ea/R*T)

Kcat _Value_ obtained **0.029 min^-1^ = 1.74 s^-1^**

Vm = 1.86 nmol/min per 20x10^6^ cells

Vm = 0.031 nmol/sec per 20.10^6^ cells

Et = Vm/kcat = 0.031/1.74 = 0.017 nmol per 20.10^6^ cells/5.4 x 10^-4^ nmol per 20.10^6^ cells/0.065 nmol per 20.10^6^ cells

= 1.7 x10^-11^/5.4 x10^-13^/6.5 x10^-11^ mol

= 8.5 x10^-12^/2.7 x10^-13^/3.25 x10^-11^ mol x 1.10^7^ cells

= 8.5x 10^-19^ = 0.85/0.027/3.25 attomol per cells

Average: 1.38 ± 1.37 attomol per cells
